# Application of patient-reported outcomes in clinical trials of traditional Chinese medicine registered in international clinical trials registry platform, from 2010 to 2022: a cross-sectional study

**DOI:** 10.1186/s41687-025-00982-2

**Published:** 2026-01-08

**Authors:** Yuanyuan Lin, Xiaowen Zhang, Zhenqian Xu, Lin Liu, Chen Shen, Mei Han, Huijuan Cao, Yutong Fei, Jianping Liu, Hongguo Rong, Chunxia Zhou

**Affiliations:** 1 Center for Evidence-Based Chinese Medicine, Beijing University of Chinese Medicine, Beijing, China; 2 Dongzhimen Hospital, Beijing University of Chinese Medicine, Beijing, China; 3 Institute for Excellence in Evidence- Based Chinese Medicine, Beijing University of Chinese Medicine, Beijing, 100029 China; 4 The Second Medical Center of PLA General Hospital, Beijing, 100853 China

**Keywords:** Traditional Chinese medicine, Patient-reported outcomes, Cross-sectional study, Clinical trials, Outcome study

## Abstract

**Purpose:**

Patient-reported outcomes (PROs) assist patients and clinicians in assessing treatment effectiveness and enhancing healthcare quality. This study aims to explore and analyze the application and characteristics of PROs in clinical trials of Traditional Chinese Medicine (TCM).

**Methods:**

This cross-sectional study was based on randomized clinical trials of TCM between January 1, 2010, and December 31, 2022 in International Clinical Trials Registry Platform. For each included trial, data including study phase, design, participant demographics, target diseases, PROs, and PRO measurements were extracted. Trials were categorized into three groups: (1) recorded specified patient-reported outcome tools, (2) referenced patient subjective outcomes without specified tools, and (3) did not mention any PROs. Further descriptive statistical analysis were conducted on the most commonly used PRO tools in different countries and for different diseases.

**Results:**

Among a total of 7783 eligible trials, 4858 (62.4%) listed explicit PRO tools, and 850 (10.9%) referenced PROs without specified tools. The most common conditions evaluated by PRO tools were musculoskeletal diseases (935 trials, 19.2%), symptoms (714, 14.7%), and neurological diseases (500, 10.3%). Frequently used PRO tools included the Visual Analogue Scale (VAS), 36-item Short-Form Health Questionnaire, and Pittsburgh Sleep Quality Index. Regionally, most PRO-related trials were in the Western Pacific (3904, 68.4%) and fewest in Africa (8, 0.1%). Countries conducting the most PRO-related trials were China, Iran, the USA, South Korea, and Brazil, focusing on musculoskeletal, symptoms, neurological, genitourinary, and digestive diseases, with varying popular disease-specific PRO tools by country. Musculoskeletal diseases were the primary focus in China, Brazil, and South Korea.

**Conclusions:**

The use of PROs in TCM clinical trials has grown during the study period. However, there was an uneven regional distribution of PRO application and a lack of standardized, reliable PRO tools tailored for TCM. Great efforts are needed to enhance the quality and promote the use of PRO tools in TCM clinical research.

**Supplementary Information:**

The online version contains supplementary material available at 10.1186/s41687-025-00982-2.

## Introduction

A well-designed and implemented clinical trial is expected to center on patients’ perspectives in healthcare research [1]. Patient-reported outcomes (PROs) include information about health conditions provided directly by patients, encompassing symptoms, functional ability, health-related quality of life, mental and cognitive states, overall well-being, and adverse symptom events [2–4]. Patient-reported outcome measures serve as standardized tools to assess PROs, facilitating the diagnosis of diseases or evaluation of health outcomes [5]. In the last decade, PROs and PRO measures have been extensively applied in randomized clinical trials of various medications or medical procedures to implement patient-centered care [6]. Several official guidelines from the US Food and Drug Administration (FDA) and European Medicines Agency (EMA) have encouraged sponsors to incorporate PROs as endpoint results in clinical trials to directly interpret the treatment meaningfulness to patients’ experience [7, 8]. From the perspective of health technology assessment (HTA) bodies, PROs can support the overall benefit-risk evaluation of new drugs and provide symptomatic adverse events [9], significantly influencing decisions related to medical product labeling claims, health policy, clinical guidelines, and national health services [10].

Traditional Chinese medicine (TCM), with its deep historical roots and extensive empirical accumulation, is widely utilized in clinical practice worldwide [11]. By April 2023, TCM had expanded to 196 countries and regions, earning international recognition for its unique benefits in disease prevention and rehabilitation [12]. TCM is characterized by two fundamental features: treatment based on pattern identification, and the holistic concept [13]. Treatment based on pattern identification requires the combination of four diagnostic methods: inspection, auscultation and olfaction, inquiry, and palpation, to collect patients’ information [13]. During inquiry, TCM practitioners typically inquire about patients’ subjective feelings, symptoms, and emotions in their daily life, similar to the essence of PROs [14]. The holistic concept in TCM means evaluating patients’ health status from a holistic perspective, highlighting the connections and unity among various parts of the body, between humans and nature, and between humans and the social environment, which are highly consistent with PROs including multiple aspects such as symptoms, quality of life, and psychological states. Accordingly, it is believed that utilizing standardized PRO tools for assessing TCM therapeutic effectiveness can promote the scientific advancement and global dissemination of TCM [15]. Despite the growing recognition and acceptance of PROs in pharmaceutical and surgical clinical trials [16–19], TCM clinical trials receive disproportionately minimal attention regarding PROs.

With the escalating global adoption of TCM, it is essential to explore the application of PROs in TCM clinical trials across different regional backgrounds. Therefore, this study set out to review the global application of PROs in randomized clinical trials of TCM to explore future directions and trends in designing and executing clinical trials of TCM.

## Methods

### Study design

The cross-sectional study involved interventional randomized clinical trials of TCM published between January 1, 2010, and December 31, 2022 worldwide, explored the application of PROs in these trials, and identified the most common PRO tools for various disease types across different countries. Data was collected from World Health Organization (WHO) International Clinical Trials Registry Platform (ICTRP). The WHO ICTRP provides access to a central database containing trial registrations provided by 20 registries. In light of these data, this study focused on these PRO measures which were frequently utilized in the conditions. This study followed the Strengthening the Reporting of Observational Studies in Epidemiology (STROBE) reporting guideline [20].

### Data collection and classification

**Fig. 1:**
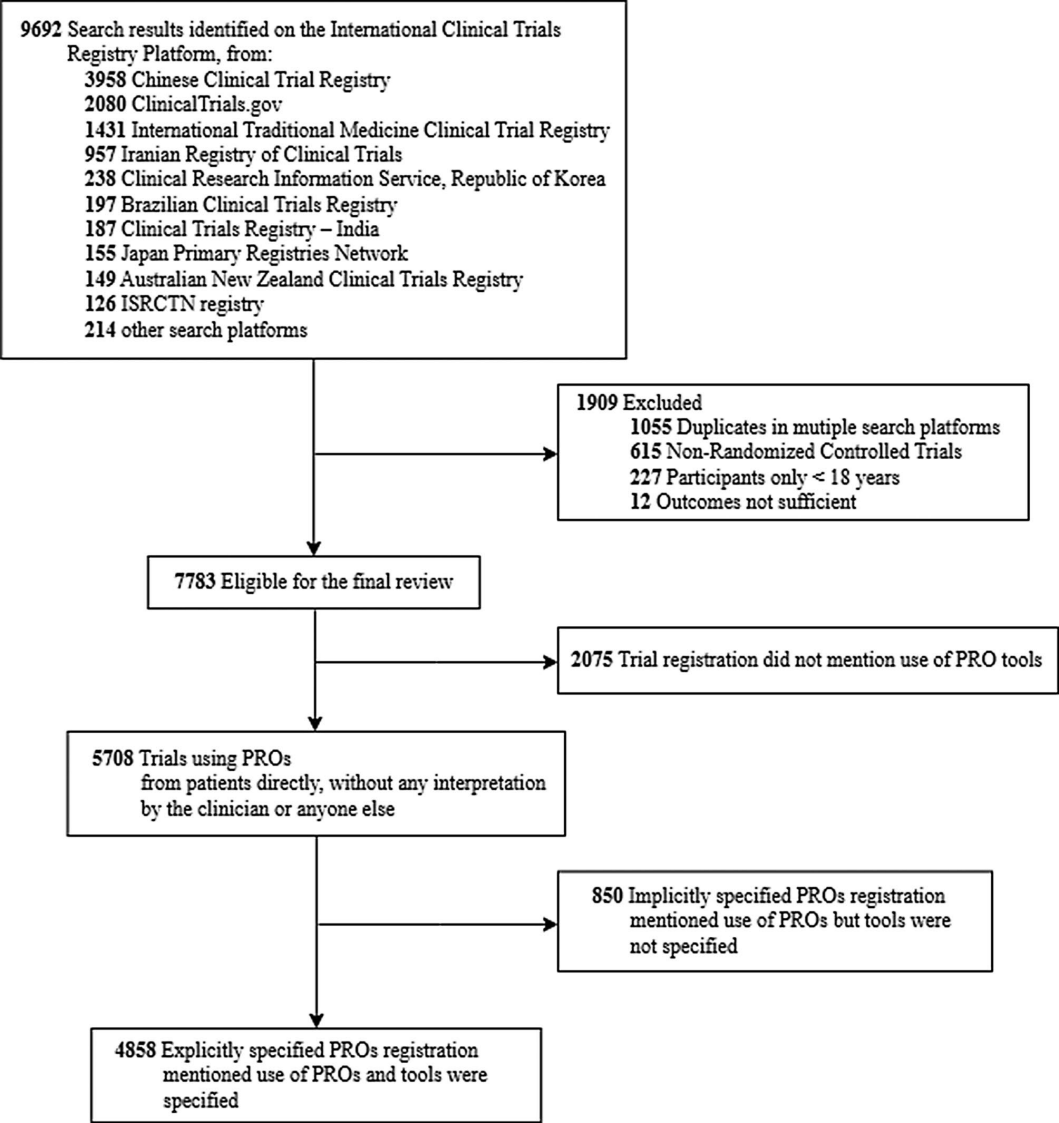
Flow diagram of trials identification and screening

This study focused on randomized clinical trials using TCM as interventional treatment (Fig. 1). We included all eligible trials, regardless of their current trial status (i.e., completed, recruiting, not yet recruiting, unknown, or terminated). Exclusion criteria were: (1) insufficient data (e.g., missing interventions or outcomes) even after verification against original registry entries; (2) duplicate registration across multiple platforms; and (3) inclusion of participants under 18 years of age. For trials that did not report the age of participants, we used general information from the trial registration platform such as study objectives, target condition, and other relevant details to infer participant age. Two authors independently reviewed the information to identify the trials meeting eligibility criteria. The discrepancies were resolved through consensus and if necessary, arbitrated by another author.

Eligible trials were categorized into three groups in accordance with published study [21]: (1) explicitly specified PROs (trial registration listed specific PRO tools), (2) implicitly specified PROs (trial registration listed PROs as outcomes, but did not mention the specific PRO tools), or (3) PROs not mentioned (trial registration did not mention any use of PRO). PRO tools were identified referring to the Electronic Patient-Reported Outcome and Validated Instruments Database for Evaluation and Research (ePROVIDE) [22]. If a PRO tool was not found in ePROVIDE but was validated in publicly published papers, it was also included.

### Data extraction

After a pilot aimed at standardizing the process, two authors independently extracted data from all included trials using a predesigned data extraction table. Discrepancies were resolved through discussion and with the involvement of a third author. The detailed information included: (1) basic information, including registration number, date of registration, official title, sponsor, recruitment status; (2) key information, including interventions, PROs, explicit PRO tools, target disease, participant age and gender; (3) study design information, including study phase, anticipated enrollment, interventional model, blinding and use of placebo.

### Statistical analysis

The trial characteristics were summarized in a descriptive manner employing counts and percentages. The Chi-square trend test was used to assess trends in the proportion of trials reporting explicitly specified PROs over time. The name and frequency of corresponding PRO tools used in each trial were recorded and calculated. Further analysis identified the most frequently used PRO tool for each disease type in PRO-related trials. To better illustrate the regional application of PRO in clinical trials, countries were grouped into six regions according to the WHO standards. Due to the diverse categories and wide variation of target conditions, similar diseases were classified into the same category following the International Classification of Diseases-11. Descriptive statistics were performed using SPSS software, version 27.0 (IBM Corp).

### Ethics approval statement

Under the Common Rule (45 CFR part 46) of the US Department of Health and Human Services, this study was exempt from institutional review board (IRB) approval and the requirement for informed patient consent. This exemption was applicable because the research did not involve clinical data or human subjects.

## Results

### Trial characteristics

**Fig. 2:**
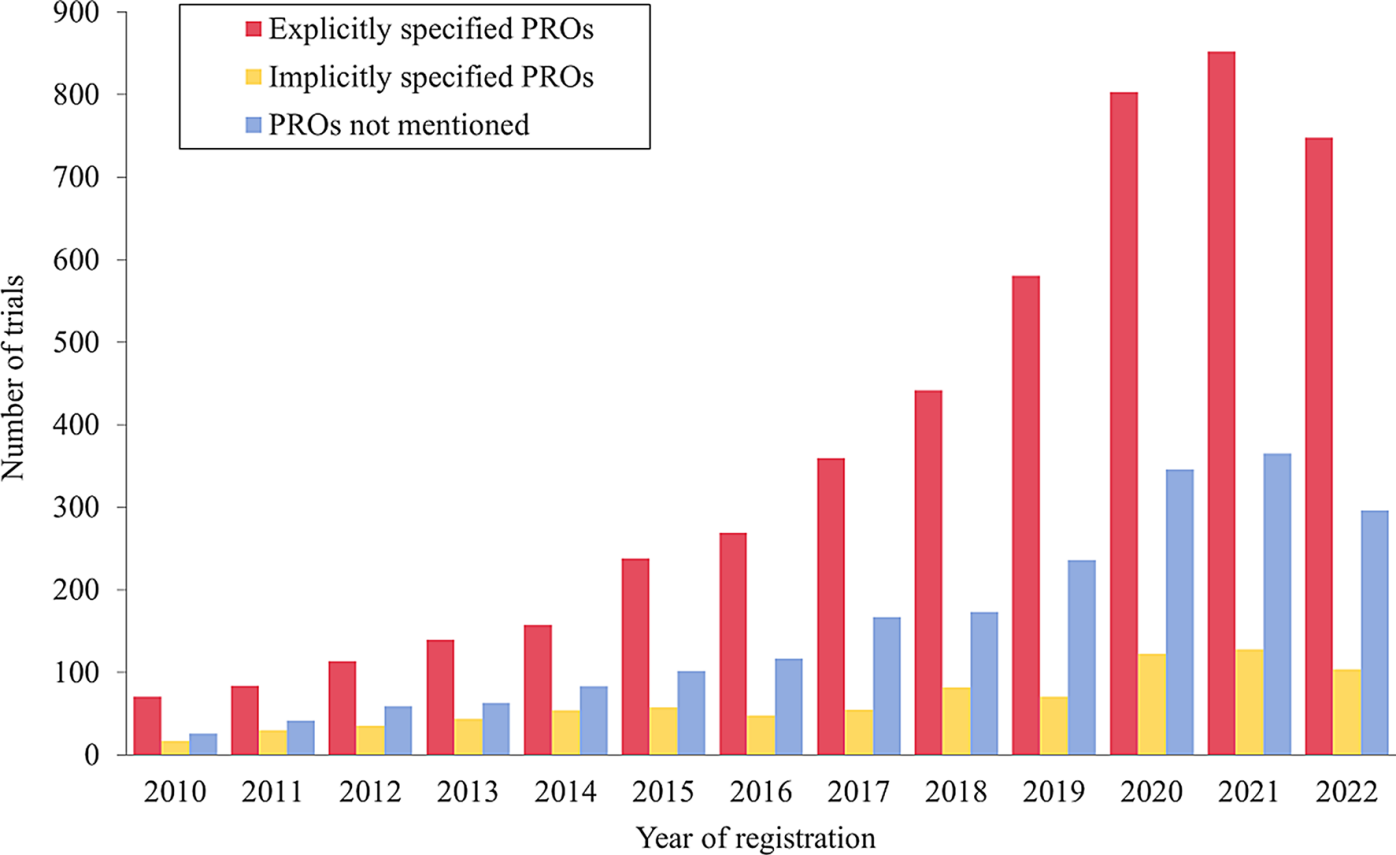
Number of clinical trials of TCM analyzed worldwide, 2010–2022

The overall characteristics of the included trials are summarized in Table 1. Figure 2 shows an increasing trend in the number of TCM clinical registration trials from 2010 to 2022 worldwide. The Chi-square trend test revealed an upward linear trend in the proportion of explicitly specified PROs over time (χ² = 22.091, *p* < 0.001), indicating the growing recognition of explicitly reported PROs in TCM trials. Among the 7783 eligible trials, 4858 (62.4%) trials listed specified PRO tools, and 850 (10.9%) did not introduce the tools that were chosen. Nearly 90% (6906, 88.7%) of the trials had no upper limit on participant age. The proportion of participants in different age groups in total trials was comparable to those involving PROs. More than 80% of the trials recruited patients of both genders. The included trials were grouped into five categories based on TCM interventions, and the majority (4975, 63.9%) focused on acupuncture therapy, followed by Chinese herbal medicines (1995, 25.6%). Similar results were observed in trials related to PRO. Approximately half of trials were in recruiting status, with only 230 (4.0%) of the trials were completed. Most primary sponsors were located in the Western Pacific region, followed by the Eastern Mediterranean region, the Americas region, the European region, and the South-East Asia region. The smallest number of trials was from the Africa region, accounting for less than 0.2% of the trials.

**Table 1:** Characteristics of all trials and trials including PRO

Characteristics		Total, No. (%)	
		Trials	PRO trials
No.		7783	5708
Age			
	18-no limit	6906 (88.7)	5099 (89.3)
	Over 65	116 (1.5)	70 (1.2)
	Unclear	761 (9.8)	539 (9.4)
Gender			
	Both	6359 (81.7)	4611 (80.8)
	Male	308 (4.0)	230 (4.0)
	Female	996 (12.8)	764 (13.4)
	Unclear	120 (1.5)	103 (1.8)
Interventions			
	Acupuncture	4975 (63.9)	3749 (65.7)
	Chinese herbal medicines	1995 (25.6)	1346 (23.6)
	Tuina (Massage)	248 (3.2)	196 (3.4)
	Qigong	227 (2.9)	171 (3.0)
	Other^a^	338 (4.3)	246 (4.3)
Primary sponsor	Hospital	3082 (39.6)	2186 (38.3)
	University	2524 (32.4)	1954 (34.2)
	Hospital and university	782 (10.0)	557 (9.8)
	Industry	151 (1.9)	103 (1.8)
	Institute	268 (3.4)	207 (3.6)
	Individual	390 (5.0)	292 (5.1)
	Government	505 (6.5)	346 (6.1)
	Other^b^	56 (0.7)	47 (0.8)
	Unclear	25 (0.3)	16 (0.3)
Recruitment status	Recruiting	3721 (47.8)	2726 (47.8)
	Not yet recruiting	3571 (45.9)	2662 (46.6)
	Completed	339 (4.4)	230 (4.0)
	Unknown status	147 (1.9)	88 (1.5)
	Terminated	5 (0.1)	2 (0.0)
Regions			
	WHO Western Pacific region	5475 (70.3)	3904 (68.4)
	WHO Eastern Mediterranean region	910 (11.7)	707 (12.4)
	WHO region of the Americas	643 (8.3)	485 (8.5)
	WHO European region	522 (6.7)	432 (7.6)
	WHO South-East Asia region	225 (2.9)	173 (3.0)
	WHO African region	8 (0.1)	7 (0.1)

^a^ Including nursing of traditional medicine, medicinal beverage and food, music therapy, and combination therapy using multiple interventions

^b^ Including foundation, government and hospital, government and industry, government and university, and hospital and industry

### Study design characteristics of registered trials

Table 2 presents the study design characteristics of the registered trials. Among the 7783 clinical trials, pilot studies and phase 4 trials were more common, representing 23.2% (*n* = 1806) and 6.8% (528) respectively. Of the 5708 PRO-related trials, pilot studies (1281, 22.4%) likewise accounted for more than phase 4 trial (359, 6.3%), with phase 1 trials accounting for the smallest percentage (183, 3.2%). A total of 97% of trials involved 500 or less participants, with 4625 (59.4%) having a sample size of less than 100. Similar findings were observed when considering both total trials and only the PRO-related trials. Nearly 93% of clinical trials used parallel design and fewer than 7% used other types of interventional models. The double-blind method was the most common form of blinding, used in 21.9% of all trials, followed by single-blind (19.9%), open-label (13.9%), and triple-blind (10.1%) designs. However, PRO-related trials were more likely to employed a single-blind design (22.1%) rather than a double-blind design (21.8%). Finally, only 2578 (33.1%) of the TCM clinical trials were placebo-controlled, likewise, just 1946 (34.1%) of the PRO-related trials were placebo-controlled.

**Table 2:** Study design characteristics of all trials and trials including PRO

Characteristics		Total, No. (%)	
		Trials	PRO trials
Study phase			
	Early stage	1806 (23.2)	1281(22.4)
	1	281 (3.6)	183(3.2)
	2	311 (4.0)	235(4.1)
	3	376 (4.8)	274(4.8)
	4	528 (6.8)	359(6.3)
	Other^a^	488 (6.3)	337(5.9)
	Unclear	3993 (51.3)	3039(53.2)
Anticipated enrollment			
	0-100	4625 (59.4)	3341(58.5)
	101–200	1946 (25.0)	1495(26.2)
	201–500	959 (12.3)	709(12.4)
	501–1000	186 (2.4)	131(2.3)
	>1000	67 (0.9)	32(0.6)
Interventional model			
	Parallel design	7215 (92.7)	5315(93.1)
	Cross-over design	248 (3.2)	155(2.7)
	Factorial design	99 (1.3)	74(1.3)
	Unclear	221 (2.8)	164(2.9)
Blinding			
	Open-label	1084 (13.9)	817(14.3)
	Single-blind	1552 (19.9)	1262(22.1)
	Double-blind	1706 (21.9)	1247(21.8)
	Triple-blind	786 (10.1)	587(10.3)
	Unclear	2655 (34.1)	1795(31.4)
Placebo			
	Yes	2578 (33.1)	1946(34.1)
	No	4124 (53.0)	3089(54.1)
	Unclear	1081 (13.9)	673(11.8)

^a^ New treatment measurements, behavioral interventions, health service, and therapeutic devices

### Target conditions and PRO tools used in clinical trials worldwide

Table 3 provides the global frequency of the use of PRO tools by target condition. Among the 4858 trials that recorded specified PRO tools, the most common conditions were musculoskeletal (935, 19.2%), symptoms (714, 14.7%), neurological (500, 10.3%), genitourinary (381, 7.8%), digestive (342, 7.0%) diseases, and mental health (329, 6.8%). Pregnancy-related diseases, skin diseases, and infectious or parasitic diseases accounted for 1% to 3% of these trials. Although the PRO tools varied by disease type, Visual Analogue Scale (VAS), 36-item Short-Form Health Questionnaire (SF-36), Pittsburgh Sleep Quality Index (PSQI) were the most frequently used tools, representing 32.9% (1600/4858), 10.1% (492/4858), and 9.3% (452/4858) of the trials, respectively. Additionally, Chinese Medicine Syndrome Score (CMSS), Numeric Rating Scale (NRS), and TCM symptom score (TCMSS) were used in 7.5% (363/4858), 6.2% (302/4858), and 5.9% (286/4858) of the trials. Trials conducted for neurological, genitourinary, digestive, circulatory, and metabolic conditions frequently focused on scales measuring TCM symptoms, such as CMSS and TCMSS. There were also specialized disease-specific PRO tools on certain conditions, such as the Western Ontario and McMaster Universities Osteoarthritis Index (WOMAC) and Oswestry Disability Index (ODI) for musculoskeletal diseases, EORTC Quality of Life Questionnaire - Core Questionnaire (EORTC QLQ-C30) for neoplasms, Seattle Angina Questionnaire (SAQ) for circulatory diseases. Interestingly, the PSQI was the chief tool to evaluate 73.4% of sleep disorders trials and 18.2% of mental health trials.

**Table 3:** Frequency of the use of PRO tools by conditions

		PRO tools									
Conditions	Proportion No. (%)	Name	No./total no. (%)	Name	No./total no. (%)	Name	No./total no. (%)	Name	No./total no. (%)	Name	No./total no. (%)
Total no.	4858										
Musculoskeletal	935 (19.2)	VAS	536/935 (57.3)	WOMAC	173/935 (18.5)	SF-36	140/935 (15.0)	ODI	89/935 (9.5)	NRS	85/935 (9.1)
Symptoms	714 (14.7)	VAS	325/714 (45.5)	NRS	75/714 (10.5)	SF-36	70/714 (9.8)	ODI	49/714 (6.9)	PSQI	42/714 (5.9)
Neurological	500 (10.3)	VAS	108/500 (21.6)	PSQI	51/500 (10.2)	SF-36	50/500 (10.0)	CMSS	37/500 (7.4)	NRS	32/500 (6.4)
Genitourinary	381 (7.8)	VAS	92/381 (24.1)	CMSS	45/381 (11.8)	TCMSS	40/381 (10.5)	SAS	30/381 (7.9)	SF-36	27/381 (7.1)
Digestive	342 (7.0)	VAS	81/342 (23.7)	CMSS	44/342 (12.9)	TCMSS	33/342 (9.6)	SF-36	32/342 (9.4)	IBS-SSS	30/342 (8.8)
Mental health	329 (6.8)	PSQI	60/329 (18.2)	VAS	40/329 (12.2)	SDS	32/329 (9.7)	SF-36	30/329 (9.1)	SAS	29/329 (8.8)
Circulatory	250 (5.1)	SAQ	58/250 (23.2)	CMSS	55/250 (22.0)	TCMSS	36/250 (14.4)	VAS	35/250 (14.0)	SF-36	26/250 (10.4)
Neoplasms	232 (4.8)	VAS	51/232 (22.0)	EORTC QLQ-C30	31/232 (13.4)	CMSS	20/232 (8.6)	TCMSS	20/232 (8.6)	PSQI	17/232 (7.3)
Respiratory	178 (3.7)	CAT	35/178 (19.7)	VAS	35/178 (19.7)	TCMSS	21/178 (11.8)	TNSS	21/178 (11.8)	mMRC/SGRQ	20/178 (11.2)
Sleep disorders	177 (3.6)	PSQI	130/177 (73.4)	ISI	64/177 (36.2)	SAS	27/177 (15.3)	SDS	25/177 (14.1)	ESS	22/177 (12.4)
Metabolic and endocrine	157 (3.2)	CMSS	36/157 (22.9)	SF-36	28/157 (17.8)	SAS	21/157 (13.4)	VAS	19/157 (12.1)	SDS	17/157 (10.8)
Pregnancy, childbirth, or the puerperium	108 (2.2)	VAS	50/108 (46.3)	NRS	13/112 (12.0)	CMSS	8/112 (7.4)	SF-36	7/112 (6.5)	MPQ	6/112 (5.6)
Skin	92 (1.9)	VAS	40/92 (43.5)	DLQI	39/92 (42.4)	TCMSS	10/92 (10.9)	CMSS	9/92 (9.8)	PSQI	7/92 (7.6)
Infectious or parasitic diseases	56 (1.2)	VAS	14/56 (25.0)	CMSS	13/56 (23.2)	NRS	6/56 (10.7)	PSQI	6/56 (10.7)	SF-36	6/56 (10.7)
Visual	45 (0.9)	OSDI	20/45 (44.4)	VAS	10/45 (22.2)	SF-36	7/45 (15.6)	NRS	7/45 (15.6)	TCMSS	4/45 (8.9)
Immune	29 (0.6)	VAS	11/29 (37.9)	HAQ	7/29 (24.1)	SF-36	7/29 (24.1)	BASDAI	4/29 (13.8)	ESSPRI	4/29 (13.8)
Ear or mastoid process	24 (0.5)	THI	12/24 (50.0)	VAS	11/24 (45.8)	PSQI	3/24 (12.5)	GAD-7	2/24 (8.3)	PHQ-9	2/24 (8.3)
Sexual health	18 (0.4)	IIEF	8/18 (44.4)	TCMSS	6/18 (33.3)	SAS	4/18 (22.2)	SDS	3/18 (16.7)	EHS	2/18 (11.1)

^a^ Symptoms, signs or clinical findings, not elsewhere classified, such as chronic pain, stress urinary incontinence, and fatigue

Abbreviations: VAS, Visual Analogue Scale; WOMAC, The Western Ontario and McMaster Universities Osteoarthritis Index; SF-36, Short-Form 36-item Health Survey; ODI, Oswestry Disability Index; NRS, Numeric Rating Scale; PSQI, Pittsburgh Sleep Quality Index; CMSS, Chinese Medicine Syndrome Score; TCMSS, TCM Symptom Score; SAS, Self-rating Anxiety Scale; IBS-SSS, Irritable Bowel Syndrome Severity Scoring System; SDS, Self-rating Depression Scale; SAQ, Seattle Angina Questionnaire; EORTC QLQ-C30, EORTC Quality of Life Questionnaire - Core Questionnaire; CAT, COPD Assessment Test; TNSS, Total Nasal Symptom Score; mMRC, modified British medical research council; SGRQ, St George’s Respiratory Questionnaire; ISI, Insomnia Severity Index; ESS, Epworth Sleepiness Scale; MPQ, McGill Pain Questionnaire; DLQI, Dermatology Life Quality Index; OSDI, Ocular Surface Disease Index; HAQ, Health Assessment Questionnaire; BASDAI, Bath Ankylosing Spondylitis Disease Activity Index; ESSPRI, EULAR Sjögren syndrome Patient-Reported Index; THI, Tinnitus Handicap Inventory; GAD-7, Generalized Anxiety Disorder − 7; PHQ-9, Patient Health Questionnaire − 9-item; IIEF, International Index of Erectile Function; EHS, Erection Hardness Score

### Target conditions and PRO tools used in China, Iran, USA, South Korea, and Brazil

The distribution of primary sponsors of trials including PROs in six regions is shown in the eFigure1 in the Supplement. At the regional level, the vast majority of trials were conducted in the Western Pacific region. The number of countries in the European region conducting PRO-related clinical trials on TCM was higher than in other regions. Among different regions, China, Iran, the USA, South Korea, and Brazil conducted the most PRO-related clinical trials on TCM, totaling 4280 trials. Among the five countries, the USA, South Korea, and Brazil preferred using acupuncture to help improve health, while China and Iran were more likely to use both Chinese herbal medicines and acupuncture (eFigure2 in the Supplement). In terms of the target conditions by TCM interventions, there were more trials in Iran using Chinese medicine to treat digestive and metabolic diseases compared with clinical trials using acupuncture. In China, more trials were using Chinese medicine to treat circulatory and respiratory diseases.

**Fig. 3:**
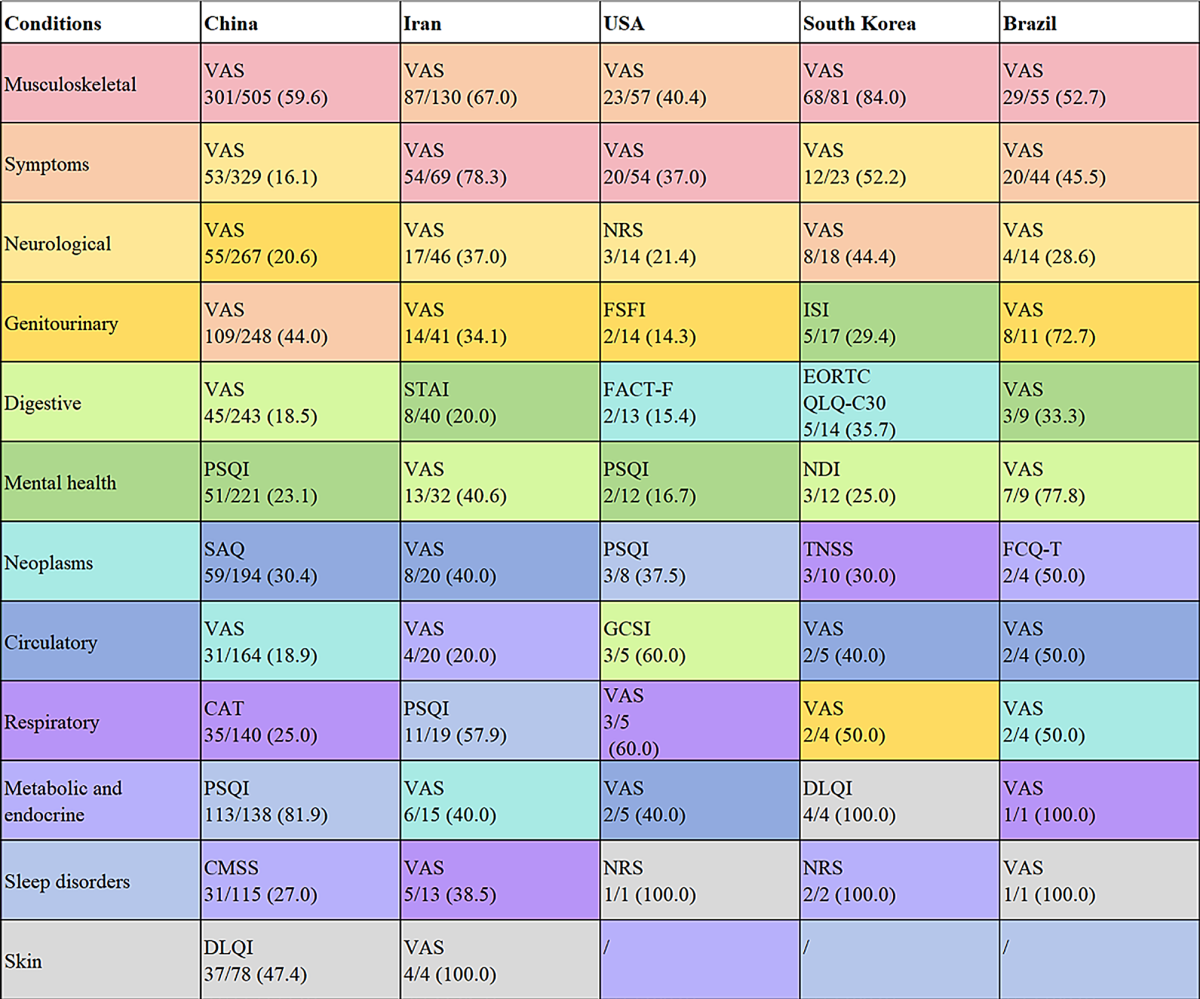
Rankings and most commonly used PRO tools of conditions in five countries

Figure 3 depicts a comprehensive overview of the rankings of target conditions and the most frequently used PRO tools in trials conducted in five countries mentioned above. Each country displayed the unique disease priorities treated with TCM and preferences for PRO tools. Notably, trials in Iran and the USA shared a focus on symptoms, musculoskeletal disorders, and neurological conditions as their primary targets for PRO assessment. Musculoskeletal diseases were the top priority in China, South Korea, and Brazil. Regarding the choice of PRO tools, significant heterogeneity was observed across the studied countries. For musculoskeletal disorders and symptom assessment, the VAS served as the primary instrument, particularly for evaluating pain outcomes. The PSQI dominated in the assessment of sleep disorders in China, Iran, and the USA, while also being employed for neurological conditions in China. For cancer, the USA and South Korea adopted disease-specific scales like the Functional Assessment of Cancer Therapy-Fatigue (FACT-F) and EORTC QLQ-C30. In other countries, VAS remained the cornerstone PRO tool. Lastly, PRO tools for mental health varied by country, incorporating instruments such as the PSQI, State-Trait Anxiety Inventory (STAI), and Insomnia Severity Index (ISI). Further analysis identified the commonly used PRO tools for musculoskeletal disorders, symptoms, and neurological conditions in five countries (eFigure3 in the Supplement).

## Discussion

This cross-sectional study investigated the application of PROs in randomized clinical trials of TCM globally. Our study found an emerging trend in the use of PROs in clinical trials of TCM from 2010 to 2022. However, merely 62.4% of the trials applied PRO tools to evaluate the outcomes directly from patients. In line with previous findings [23], the application of standard PRO tools for TCM was insufficient.

The integration of PROs in trials not only accords with the holistic philosophy of TCM but also strengthens its scientific credibility. PROs capture dimensions of health—such as fatigue, sleep quality, and emotional well-being—that are often central to TCM treatment goals yet may be overlooked by conventional endpoints [14]. Furthermore, PROs provide critical insights in contexts where objective biomarkers fail to fully capture clinical improvements, frequently observed in TCM clinical scenarios [24]. By incorporating standardized PRO tools, TCM trials can generate more comprehensive evidence, supporting broader acceptance within evidence-based medicine and facilitating cross-cultural dialogue in integrative healthcare [25].

Musculoskeletal disease represented the highest number of clinical trials including PRO items globally, especially in China, Brazil, and Korea. In 2020, musculoskeletal disorders were the second leading cause of non-fatal disability and involved more than 1.6 billion people globally [26]. Many unique TCM interventions have been validated for their efficacy in alleviating pain and enhancing physical functionality in musculoskeletal disorders [27–29]. Musculoskeletal disorders are often associated with psychological symptoms such as anxiety and depression [30], and the use of PROs and PRO tools can help assess the severity of physical and mental health [31]. Meanwhile, trials for sleep disorders centered on the application of PRO tools, with the highest PRO tools usage rate among all the trials. Increasing concern about the impact on function and quality of life from sleep disorder has inspired the development of PRO instruments [32].Our study identified that the PSQI was the most frequently used instrument of sleep quality in clinical settings. Given the widespread occurrence of sleep disturbances within individuals with mental health conditions [33], the PSQI likewise exhibits extensive applicability in the domain of mental health clinical practices [34].

VAS was found to be the most common tool in included trials. On the one hand, the value of VAS is continuously changing which can reflect the subtle changes in symptoms better than the graded options. On the other hand, statistically, continuous score is convenient for parameter testing in precise comparison of outcomes between groups. VAS was initially applied to assess the intensity and the affective magnitude of pain [35, 36],but recently has been widely used to measure other symptoms, mental states and psychological well-being [37–40]. To carry out objective and accurate quantification in the evaluation of TCM treatment, we suggest using VAS to measure the core symptoms of TCM which target the center of the disease or the main therapeutic objectives. There were differences in choosing disease-specific PRO tools in China, Iran, the USA, Korea, and Brazil. For example, the WOMAC, a reliable tool to assess osteoarthritis-related disability in hip and knee [41], was widely used in China, Brazil, and Iran. In addition to a high incidence of osteoarthritis within the regions [42], other factors such as cross-cultural adaptation and validation of tools might influence the popularity of PRO measures among countries [43, 44]. It’s indeed intriguing that the USA demonstrated a unique preference for condition-specific PRO tools like Fibromyalgia Impact Questionnaire. Fibromyalgia is a chronic pain condition that can cause widespread pain, physical exhaustion, and cognitive difficulties [45], leading to a significant economic burden to American healthcare systems [46]. Taichi and acupuncture can be considered worthy therapeutic options in the multidisciplinary management of fibromyalgia [47–49].

Another positive sign is that some researchers have begun to pay attention to the development and evaluation of TCM-related PRO scales [50–52]. The TCMSS and CMSS were used in multiple TCM interventions to evaluate the subjects’ symptoms of various conditions [53–55]. However, in cases where questionnaire items are inconsistent, utilizing TCMSS or CMSS as the outcome measures poses challenges for comparing and evaluating the efficacy among similar clinical studies. In the process of data extraction, we also identified that some trials used PRO tools to assess patient’s perceived credibility or feelings about acupuncture, such as the Massachusetts General Hospital Acupuncture Sensation Scale, Treatment Credibility Scale, and Acupuncture Belief Scale [56, 57]. Further studies are warranted to construct more standardized and practical PRO scales suitable for TCM. Taking the Chinese Quality of Life Instrument as an example, a standardized TCM-specific PRO tool should meet adhere to several key principles: first, its items should align with the conceptual understanding of health and disease in TCM; second, it should implement multi-stage feedback mechanisms involving diverse stakeholders including TCM clinicians, patients, and pharmaceutical experts to iteratively refine draft items for content relevance and clinical sensibility; third, extensive pilot testing in representative samples is required to perform confirmatory factor analyses, evaluate structural fitness, internal consistency and discriminant validity [58]. Furthermore, distribution-based, anchor-based methods or qualitative methods can be employed to estimate responsiveness, which reflects the ability to correlating changes in the PRO scale score against changes in disease status [59].

PRO-related trials demonstrated notable variation in their study designs, including study phase, randomization methods, blinding and interventional model. A significant number of PRO-related trials are found to be conducted in the early stage of clinical trials, which can provide insight into how new interventions interact with the human body and their safety profile [60]. The inclusion of PROs in early-stage trials also enhances PRO strategy in later trial phases and may improve data collection quality for analysis [60]. Approximately 70% of PRO-related trials implemented blinding methods and 14.3% were conducted as open-label trials, a factor that could potentially contribute to a reduced rate of completion in reporting PROs in comparison to double-blind trials [61]. Besides, we noticed that PRO-related trials were more likely to use single-blind rather than double-blind compared with overall trials. Considering bias from doctor–patient interactions and subconscious cues from researchers [62], researchers should take note of double-blind methods for collecting subjective outcomes in clinical trials. A large fraction of the registered trials had a small sample size with an enrollment of less than 100 participants. Small trials may be suitable in the early phase for future sample size evaluation, but in terms of clinical trials for treatment, small trials are likely to be less informative and convincible in clinical curative effects evaluation [63]. It is recommended that future studies hold collaborative efforts across multiple centers to address the deficiency in trials of large sample size. In 2018, Guidelines for Inclusion of Patient-Reported Outcomes in Clinical Trial Protocols was published [64], aimed at promoting the scientific and rigorous trial design focus on PROs, which may serve as a directive for study designers seeking to incorporate PROs into their researches.

Not surprisingly, there were obvious regional differences in the distribution of TCM randomized clinical trials. The majority of clinical trials were in the Western Pacific region where TCM originated and developed initially. Secondly, the number of TCM trials in the Eastern Mediterranean region, region of the Americas, and European region accounted for a high proportion. This may be because the trade of Chinese medicinal materials between China and the countries along the Silk Road has promoted the spread and development of TCM overseas [65, 66].

The global application of PROs remains an evolving practice that demands constant refinement and adaptation. While PROs can inform clinical decision-making and assess treatment effectiveness, existing PRO tools may fail to capture the multidimensional and holistic effects of TCM. In this context, researchers and clinicians in the global TCM community should prioritize the development of PRO tools that are fit-for-purpose and grounded in the unique theoretical and clinical principles of TCM. A PRO instrument is considered fit-for-purpose when sufficient evidence supports the interpretability of its scores within a specific context of use [67]. To ensure applicability across multicultural settings, such tools must undergo not only linguistic translation but also cultural adaptation and validation to capture clinically meaningful changes. By advancing high-quality TCM clinical trials that incorporate PROs, we can promote more patient-centered and holistic healthcare practices worldwide.

### Limitation

There are still several limitations. Firstly, considering young children may not express their feelings accurately, and parents-reported outcomes could be influenced by multi-factors, we did not include studies of children to avoid the potential bias of the results. Secondly, although the ICTRP contains registration information from 20 registries around the world, this study may have captured more Chinese trials than those conducted elsewhere. To better illustrate the regional application of PROs worldwide and address potential geographic bias, we further analyzed the use of PROs and PRO tools across the five countries with the largest number of trials in our sample. This approach helped contextualize the overrepresentation of Chinese trials and explore regional variations in PRO adoption patterns. Finally, some registration information of trials was not updated promptly on the registry platforms. As trial characteristics were collected directly from the registry platforms, inaccuracies in the information reported by investigators may have affected the accuracy of our data and findings.

## Conclusion

This cross-sectional study indicates that PRO-related clinical trials of TCM have grown over recent years. Inadequate application of PROs and the lack of standardized, TCM-specific PRO tools remain key challenges. Future research should focus on developing validated PRO tools that reflect the unique theoretical and clinical principles of TCM. Given the uneven geographic distribution of PRO application, fit-for-purpose PRO tools developed through cultural adaptation and validation are also needed.

## Supplementary Information

Below is the link to the electronic supplementary material.

Supplementary Material 1

## Data Availability

The datasets presented in this study can be found in online repositories. Further information could be acquired by contacting with the corresponding author.

## References

[1] Calvert M, Kyte D, Von Hildebrand M, King M, Moher D (2015) Putting patients at the heart of health-care research. Lancet 385(9973):1073–1074. 10.1016/S0140-6736(15)60599-2 25797557 10.1016/S0140-6736(15)60599-2

[2] Casey DE (2022) Patient-Reported outcome Measures-Challenges and opportunities for China. JAMA Netw Open 5(5):e2211652. 10.1001/jamanetworkopen.2022.11652 35544140 10.1001/jamanetworkopen.2022.11652

[3] Basch E, Jia X, Heller G et al (2009) Adverse symptom event reporting by patients vs clinicians: relationships with clinical outcomes. J Natl Cancer Inst 101(23):1624–1632. 10.1093/jnci/djp386 19920223 10.1093/jnci/djp386 PMC2786917

[4] U.S. Department of Health and Human Services FDA Center for Drug Evaluation and Research, U.S. Department of Health and Human Services FDA Center for Biologics Evaluation and Research, U.S. Department of Health and Human Services FDA Center for Devices and Radiological Health (2006) Guidance for industry: patient-reported outcome measures: use in medical product development to support labeling claims: draft guidance. Health Qual Life Outcomes 4:79. 10.1186/1477-7525-4-79 17034633 10.1186/1477-7525-4-79 PMC1629006

[5] Gibbons C, Porter I, Gonçalves-Bradley DC et al (2021) Routine provision of feedback from patient-reported outcome measurements to healthcare providers and patients in clinical practice. Cochrane Database Syst Rev 2021(10):CD011589. 10.1002/14651858.CD011589.pub2 10.1002/14651858.CD011589.pub2 PMC8509115 34637526

[6] Winkelmann C, Mezentseva A, Vogt B, Neumann T (2023) Patient-Reported outcome measures in liver and Gastrointestinal cancer randomized controlled trials. Int J Environ Res Public Health 20(13):6293. 10.3390/ijerph20136293 37444140 10.3390/ijerph20136293 PMC10341660

[7] Research C (2023) for DE and. Patient-focused drug development: incorporating clinical outcome assessments into endpoints for regulatory decision-making. May 4, Accessed August 6, 2025. https://www.fda.gov/regulatory-information/search-fda-guidance-documents/patient-focused-drug-development-incorporating-clinical-outcome-assessments-endpoints-regulatory

[8] Pignatti F, Mol P, Quinten C et al (2025) Use of patient-reported outcomes to inform symptom and functional outcomes in cancer drug regulatory decisions: challenges and future directions. Lancet Oncol 26(6):664–666. 10.1016/S1470-2045(25)00151-2 40245905 10.1016/S1470-2045(25)00151-2

[9] Pe M, Voltz-Girolt C, Bell J et al (2025) Using patient-reported outcomes and health-related quality of life data in regulatory decisions on cancer treatment: highlights from an EMA-EORTC workshop. Lancet Oncol 26(6):687–690. 10.1016/S1470-2045(25)00150-0 40245904 10.1016/S1470-2045(25)00150-0

[10] Calvert M, King M, Mercieca-Bebber R et al (2021) SPIRIT-PRO extension explanation and elaboration: guidelines for inclusion of patient-reported outcomes in protocols of clinical trials. BMJ Open 11(6):e045105. 10.1136/bmjopen-2020-045105 34193486 10.1136/bmjopen-2020-045105 PMC8246371

[11] Cyranoski D (2018) Why Chinese medicine is heading for clinics around the world. Nature 561(7724):448–450. 10.1038/d41586-018-06782-7 30258149 10.1038/d41586-018-06782-7

[12] Official (2024) Traditional Chinese medicine spreads to 196 countries, regions | english.scio.gov.cn. Accessed February 11. http://english.scio.gov.cn/pressroom/2023-04/20/content_85242111.htm

[13] Zhong Y, Deng Y, Chen Y, Chuang PY, Cijiang He J (2013) Therapeutic use of traditional Chinese herbal medications for chronic kidney diseases. Kidney Int 84(6):1108–1118. 10.1038/ki.2013.276 23868014 10.1038/ki.2013.276 PMC3812398

[14] Zhang YH, Lv J, Gao W et al (2017) Practitioners’ perspectives on evaluating treatment outcomes in traditional Chinese medicine. BMC Complement Altern Med 17(1):269. 10.1186/s12906-017-1746-8 28521826 10.1186/s12906-017-1746-8 PMC5437539

[15] Jiang M, Yang J, Zhang C et al (2010) Clinical studies with traditional Chinese medicine in the past decade and future research and development. Planta Med 76(17):2048–2064. 10.1055/s-0030-1250456 20979016 10.1055/s-0030-1250456

[16] Mark DB, Patel MR (2017) Patient-Reported outcomes in revascularization decisions for Left-Main disease: sharing the excellence. J Am Coll Cardiol 70(25):3123–3126. 10.1016/j.jacc.2017.10.059 29097295 10.1016/j.jacc.2017.10.059

[17] Pitt SC (2022) Quality of Life, Patient-Reported Outcomes, and extent of surgery for patients with Low- and Intermediate-Risk-Differentiated thyroid cancer. JAMA Surg 157(3):209–210. 10.1001/jamasurg.2021.6443 34935879 10.1001/jamasurg.2021.6443

[18] Jim HSL, Brady-Nicholls R, Hershman DL (2023) The importance of patient-reported outcomes in pragmatic clinical trials. JNCI: J Natl Cancer Inst 115(4):352–354. 10.1093/jnci/djad037 36805254 10.1093/jnci/djad037 PMC10086620

[19] Morton LM, Hamilton BK (2020) Using patient-reported outcomes to improve survivorship care. Blood 135(21):1819–1820. 10.1182/blood.2020005881 32437561 10.1182/blood.2020005881 PMC7243152

[20] Cuschieri S (2019) The STROBE guidelines. Saudi J Anaesth 13(Suppl 1):S31–S34. 10.4103/sja.SJA_543_18 30930717 10.4103/sja.SJA_543_18 PMC6398292

[21] Zhou H, Yao M, Gu X et al (2022) Application of Patient-Reported outcome measurements in clinical trials in China. JAMA Netw Open 5(5):e2211644. 10.1001/jamanetworkopen.2022.11644 35544134 10.1001/jamanetworkopen.2022.11644 PMC9096600

[22] ePROVIDE™ - Online support for clinical outcome assessments. ePROVIDE - Mapi research trust. Accessed March 12 (2024) https://eprovide.mapi-trust.org/advanced-search

[23] Dong Y, Liu L, Zhang X et al (2023) A cross-sectional study on the application of patient-reported outcome measurements in clinical trials of traditional Chinese medicine in Mainland China. Front Pharmacol 14:1159906. 10.3389/fphar.2023.1159906 37251323 10.3389/fphar.2023.1159906 PMC10213936

[24] Yang J, Li Y, Chau CI et al (2023) Efficacy and safety of traditional Chinese medicine for cancer-related fatigue: a systematic literature review of randomized controlled trials. Chin Med 18(1):142. 10.1186/s13020-023-00849-y 37907925 10.1186/s13020-023-00849-y PMC10619240

[25] Cruz Rivera S, McMullan C, Jones L, Kyte D, Slade A, Calvert M (2020) The impact of patient-reported outcome data from clinical trials: perspectives from international stakeholders. J Patient-rep Outcomes 4(1):51. 10.1186/s41687-020-00219-4 32617713 10.1186/s41687-020-00219-4 PMC7332593

[26] GBD 2021 Other Musculoskeletal Disorders Collaborators (2023) Global, regional, and National burden of other musculoskeletal disorders, 1990–2020, and projections to 2050: a systematic analysis of the global burden of disease study 2021. Lancet Rheumatol 5(11):e670–e682. 10.1016/S2665-9913(23)00232-1 37927903 10.1016/S2665-9913(23)00232-1 PMC10620749

[27] Bervoets DC, Luijsterburg PAJ, Alessie JJN, Buijs MJ, Verhagen AP (2015) Massage therapy has short-term benefits for people with common musculoskeletal disorders compared to no treatment: a systematic review. J Physiother 61(3):106–116. 10.1016/j.jphys.2015.05.018 26093806 10.1016/j.jphys.2015.05.018

[28] Ye Z, Liu Y, Song J et al (2023) Expanding the therapeutic potential of salvia miltiorrhiza: a review of its Pharmacological applications in musculoskeletal diseases. Front Pharmacol 14:1276038. 10.3389/fphar.2023.1276038 38116081 10.3389/fphar.2023.1276038 PMC10728493

[29] Peng Z, Xu R, You Q (2022) Role of traditional Chinese medicine in bone regeneration and osteoporosis. Front Bioeng Biotechnol 10:911326. 10.3389/fbioe.2022.911326 35711635 10.3389/fbioe.2022.911326 PMC9194098

[30] Zhang W, Singh SP, Clement A, Calfee RP, Bijsterbosch JD, Cheng AL (2023) Improvements in physical function and pain interference and changes in mental health among patients seeking musculoskeletal care. JAMA Netw Open 6(6). 10.1001/jamanetworkopen.2023.20520 10.1001/jamanetworkopen.2023.20520 PMC10308248 37378984

[31] Hirao K, Takahashi H, Kuroda N, Uchida H, Tsuchiya K, Kikuchi S (2023) Differences in center for epidemiologic studies depression scale, generalized anxiety Disorder-7 and Kessler screening scale for psychological distress scores between smartphone version versus paper version administration: evidence of equivalence. Int J Environ Res Public Health 20(6):4773. 10.3390/ijerph20064773 36981682 10.3390/ijerph20064773 PMC10049019

[32] Medarov BI, Victorson DE, Judson MA (2013) Patient-reported outcome measures for sleep disorders and related problems: clinical and research applications. Chest 143(6):1809–1818. 10.1378/chest.12-2489 23732593 10.1378/chest.12-2489

[33] Buysse DJ, Reynolds CF, Monk TH, Berman SR, Kupfer DJ (1989) The Pittsburgh sleep quality index: A new instrument for psychiatric practice and research. Psychiatry Res 28(2):193–213. 10.1016/0165-1781(89)90047-4 2748771 10.1016/0165-1781(89)90047-4

[34] Doi Y, Minowa M, Uchiyama M et al (2000) Psychometric assessment of subjective sleep quality using the Japanese version of the Pittsburgh sleep quality index (PSQI-J) in psychiatric disordered and control subjects. Psychiatry Res 97(2–3):165–172. 10.1016/s0165-1781(00)00232-8 11166088 10.1016/s0165-1781(00)00232-8

[35] Huskisson EC (1974) Measurement of pain. Lancet 2(7889):1127–1131. 10.1016/s0140-6736(74)90884-8 4139420 10.1016/s0140-6736(74)90884-8

[36] Price DD, McGrath PA, Rafii A, Buckingham B (1983) The validation of visual analogue scales as ratio scale measures for chronic and experimental pain. Pain 17(1):45–56. 10.1016/0304-3959(83)90126-4 6226917 10.1016/0304-3959(83)90126-4

[37] Kasparbauer AM, Petrovsky N, Schmidt PM et al (2019) Effects of nicotine and Atomoxetine on brain function during response Inhibition. Eur Neuropsychopharmacol 29(2):235–246. 10.1016/j.euroneuro.2018.12.004 30552041 10.1016/j.euroneuro.2018.12.004

[38] Mizuno M, Fukunaga A, Washio K, Imamura S, Oda Y, Nishigori C (2020) A visual analogue scale for itch and pain in 23 cases of cholinergic urticaria. J Eur Acad Dermatol Venereol 34(9):e493–e495. 10.1111/jdv.16410 32242985 10.1111/jdv.16410

[39] Moor CC, Mostard RLM, Grutters JC, Bresser P, Wijsenbeek MS (2022) The use of online visual analogue scales in idiopathic pulmonary fibrosis. Eur Respir J 59(1):2101531. 10.1183/13993003.01531-2021 34326190 10.1183/13993003.01531-2021 PMC8756292

[40] Bengtsson M, Ohlsson B (2013) The brief visual analogue scale for irritable bowel syndrome questionnaire can be used to evaluate psychological well-being in patients with irritable bowel syndrome. Eur J Intern Med 24(7):e82–e83. 10.1016/j.ejim.2013.05.013 23773543 10.1016/j.ejim.2013.05.013

[41] McConnell S, Kolopack P, Davis AM (2001) The Western Ontario and McMaster universities osteoarthritis index (WOMAC): a review of its utility and measurement properties. Arthritis Care Res 45(5):453–461. 10.1002/1529-0131(200110)45:5%3C453:AID-ART365%3E;3.0.CO;2-W. 10.1002/1529-0131(200110)45:5<453::aid-art365>3.0.co;2-w 11642645

[42] Global regional (2018) National incidence, prevalence, and years lived with disability for 354 diseases and injuries for 195 countries and territories, 1990–2017: a systematic analysis for the global burden of disease study 2017. Lancet 392(10159):1789–1858. 10.1016/S0140-6736(18)32279-7 30496104 10.1016/S0140-6736(18)32279-7 PMC6227754

[43] Ferreira C, de SB, Dibai-Filho AV, Almeida DO da (2020) Structural validity of the Brazilian version of the Western Ontario and McMaster universities osteoarthritis index among patients with knee osteoarthritis. Sao Paulo Med J 138(5):400–406. 10.1590/1516-3180.2020.0046.R1.26062020 10.1590/1516-3180.2020.0046.R1.26062020 PMC9673866 33084741

[44] Bae SC, Lee HS, Yun HR, Kim TH, Yoo DH, Kim SY (2001) Cross-cultural adaptation and validation of Korean Western Ontario and McMaster universities (WOMAC) and Lequesne osteoarthritis indices for clinical research. Osteoarthritis Cartilage 9(8):746–750. 10.1053/joca.2001.0471 11795994 10.1053/joca.2001.0471

[45] Häuser W, Ablin J, Fitzcharles MA et al (2015) Fibromyalgia. Nat Rev Dis Primers 1:15022. 10.1038/nrdp.2015.22 27189527 10.1038/nrdp.2015.22

[46] D’Onghia M, Ciaffi J, Ruscitti P et al (2022) The economic burden of fibromyalgia: A systematic literature review. Semin Arthritis Rheum 56:152060. 10.1016/j.semarthrit.2022.152060 35849890 10.1016/j.semarthrit.2022.152060

[47] Goldenberg DL, Burckhardt C, Crofford L (2004) Management of fibromyalgia syndrome. JAMA 292(19):2388–2395. 10.1001/jama.292.19.2388 15547167 10.1001/jama.292.19.2388

[48] Deluze C, Bosia L, Zirbs A, Chantraine A, Vischer TL (1992) Electroacupuncture in fibromyalgia: results of a controlled trial. BMJ 305(6864):1249–1252 1477566 10.1136/bmj.305.6864.1249 PMC1883744

[49] Wang C, Schmid CH, Fielding RA et al (2018) Effect of Tai Chi versus aerobic exercise for fibromyalgia: comparative effectiveness randomized controlled trial. BMJ 360:k851. 10.1136/bmj.k851 29563100 10.1136/bmj.k851 PMC5861462

[50] Zhao L, Chan K (2005) Building a Bridge for integrating Chinese medicine into conventional healthcare: observations drawn from the development of the Chinese quality of life instrument. Am J Chin Med 33(6):897–902. 10.1142/S0192415X05003533 16355446 10.1142/S0192415X05003533

[51] Bai MH, Li ZQ, Wang HY et al (2023) Development and evaluation of short-form version of the constitution in Chinese medicine questionnaire: study a new and best brief instrument of Chinese medicine for health management. Chin Med 18(1):140. 10.1186/s13020-023-00844-3 37904166 10.1186/s13020-023-00844-3 PMC10617149

[52] Sun L, Mao JJ, Yan Y, Xu Y, Yang Y (2021) Patient reported traditional Chinese medicine spleen deficiency syndrome (TCM-SDS) scale for colorectal cancer: development and validation in China. Integr Cancer Ther 20:15347354211020105. 10.1177/15347354211020105 34116615 10.1177/15347354211020105 PMC8202331

[53] Xu DP, Wu HL, Lan TH et al (2015) Effect of Shenzhu Guanxin recipe () on patients with angina pectoris after percutaneous coronary intervention: A prospective, randomized controlled trial. Chin J Integr Med 21(6):408–416. 10.1007/s11655-015-2040-6 26063318 10.1007/s11655-015-2040-6

[54] Zhang Y, Xu L, Wang X et al (2023) The clinical efficacy of Shenxiang Suhe pill administration in posterior circulation ischemic vertigo: A randomized controlled trial. Med (Baltim) 102(51):e36604. 10.1097/MD.0000000000036604 10.1097/MD.0000000000036604 PMC10735062 38134060

[55] Pan J, Xu Y, Chen S et al (2021) The effectiveness of traditional Chinese medicine Jinlida granules on glycemic variability in newly diagnosed type 2 diabetes: A Double-Blinded, randomized trial. J Diabetes Res 2021:6303063. 10.1155/2021/6303063 34660811 10.1155/2021/6303063 PMC8519714

[56] Cui H, Yu H, Huang X et al (2021) Electroacupuncture and transcutaneous electrical nerve stimulation induced sensations in bell’s palsy patients: A quantitative current intensity analysis. Front Neurosci 15:692088. 10.3389/fnins.2021.692088 34305521 10.3389/fnins.2021.692088 PMC8299110

[57] Chae Y, Kim SY, Park HS, Lee H, Park HJ (2008) Experimentally manipulating perceptions regarding acupuncture elicits different responses to the identical acupuncture stimulation. Physiol Behav 95(3):515–520. 10.1016/j.physbeh.2008.07.027 18725240 10.1016/j.physbeh.2008.07.027

[58] LeungKfai, bin Liu F, Zhao L, Fang Jqian, Chan K, Lin L zhu (2005). Development and validation of the Chinese quality of life instrument. Health Qual Life Outcomes. 3:26. 10.1186/1477-7525-3-26 10.1186/1477-7525-3-26 PMC1090607 15833138

[59] Kosinski M, Nelson LM, Stanford RH, Flom JD, Schatz M (2024) Patient-reported outcome measure development and validation: a primer for clinicians. J Allergy Clin Immunol Pract 12(10):2554–2561. 10.1016/j.jaip.2024.08.030 39181327 10.1016/j.jaip.2024.08.030

[60] Retzer A, Aiyegbusi OL, Rowe A et al (2022) The value of patient-reported outcomes in early-phase clinical trials. Nat Med 28(1):18–20. 10.1038/s41591-021-01648-4 35039659 10.1038/s41591-021-01648-4

[61] Roydhouse JK, King-Kallimanis BL, Howie LJ, Singh H, Kluetz PG (2019) Blinding and Patient-Reported outcome completion rates in US food and drug administration cancer trial Submissions, 2007–2017. J Natl Cancer Inst 111(5):459–464. 10.1093/jnci/djy181 30561711 10.1093/jnci/djy181

[62] Brody I (1973) Double-blind or single-blind? N Engl J Med 288(5):267–268. 10.1056/NEJM197302012880519 4565762 10.1056/NEJM197302012880519

[63] Califf RM, Zarin DA, Kramer JM, Sherman RE, Aberle LH, Tasneem A (2012) Characteristics of clinical trials registered in ClinicalTrials.gov, 2007–2010. JAMA 307(17):1838–1847. 10.1001/jama.2012.3424 22550198 10.1001/jama.2012.3424 PMC13431740

[64] Calvert M, Kyte D, Mercieca-Bebber R et al (2018) Guidelines for inclusion of Patient-Reported outcomes in clinical trial protocols: the SPIRIT-PRO extension. JAMA 319(5):483–494. 10.1001/jama.2017.21903 29411037 10.1001/jama.2017.21903

[65] Tang K, Li Z, Li W, Chen L (2017) China’s silk road and global health. Lancet 390(10112):2595–2601. 10.1016/S0140-6736(17)32898-2 29231838 10.1016/S0140-6736(17)32898-2 PMC7159269

[66] Shi J, Yang Y, Zhou X et al (2022) The current status of old traditional medicine introduced from Persia to China. Front Pharmacol 13:953352. 10.3389/fphar.2022.953352 36188609 10.3389/fphar.2022.953352 PMC9515588

[67] FDA-NIH Biomarker Working Group. BEST (Biomarkers, EndpointS, and Other Tools) Resource, Food, Administration D, US) (2016) (; Accessed August 6, 2025. http://www.ncbi.nlm.nih.gov/books/NBK326791/

