# Supplementary material for: Application of patient-reported outcomes in clinical trials of traditional Chinese medicine registered in international clinical trials registry platform, from 2010 to 2022: a cross-sectional study
Source: J Patient Rep Outcomes. 2026 Jan 8;10:18. doi: 10.1186/s41687-025-00982-2 (PMC12881188; doi:10.1186/s41687-025-00982-2)
Supplement: Supplementary file 1 — Supplementary Material 1 [file 41687_2025_982_MOESM1_ESM.pdf]

Supplementary Online Content

eFigure 1. Number of trials with PROs in each region and country.

eFigure 2. Number of trials with PROs using Chinese herbal medicines or acupuncture in five countries by conditions.

eFigure 3. The top three target conditions and frequently used PRO tools across five countries

This supplementary material has been provided by the authors to give readers additional information about their work.

eFigure 1. Number of trials with PROs in each region and country.
(Countries that conducted PRO-related randomized clinical trials less than 0.1% of the total were not shown in the figure.)

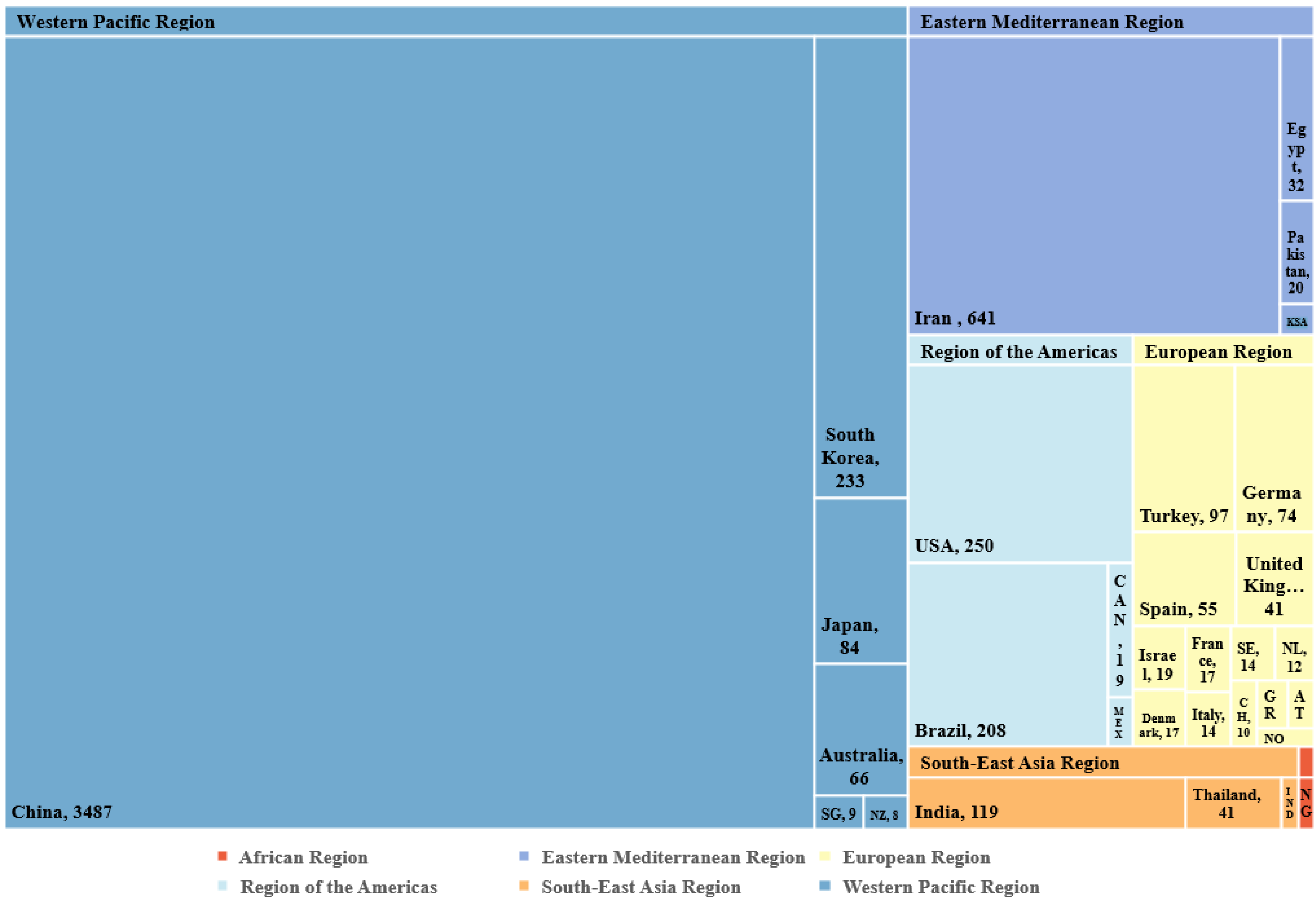

Abbreviations: KSA, Saudi Arabia; CAN, Canada; MEX, Mexico; SG, Singapore; NZ, New Zealand; SE, Sweden; NL, Netherlands; CH, Switzerland; GR, Greece, AT, Austria; NO, Norway; IND, Indonesia; NG, Nigeria.

eFigure 2. Number of trials with PROs using Chinese herbal medicines or acupuncture in five countries by conditions.

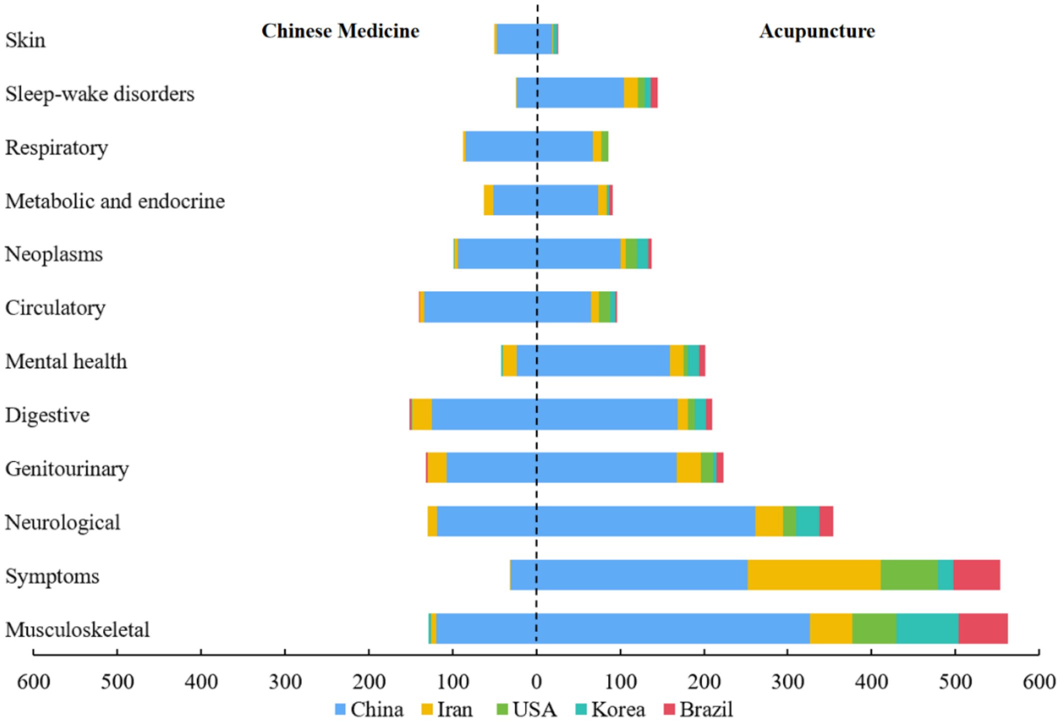

eFigure 3. The top three target conditions and frequently used PRO tools across five countries

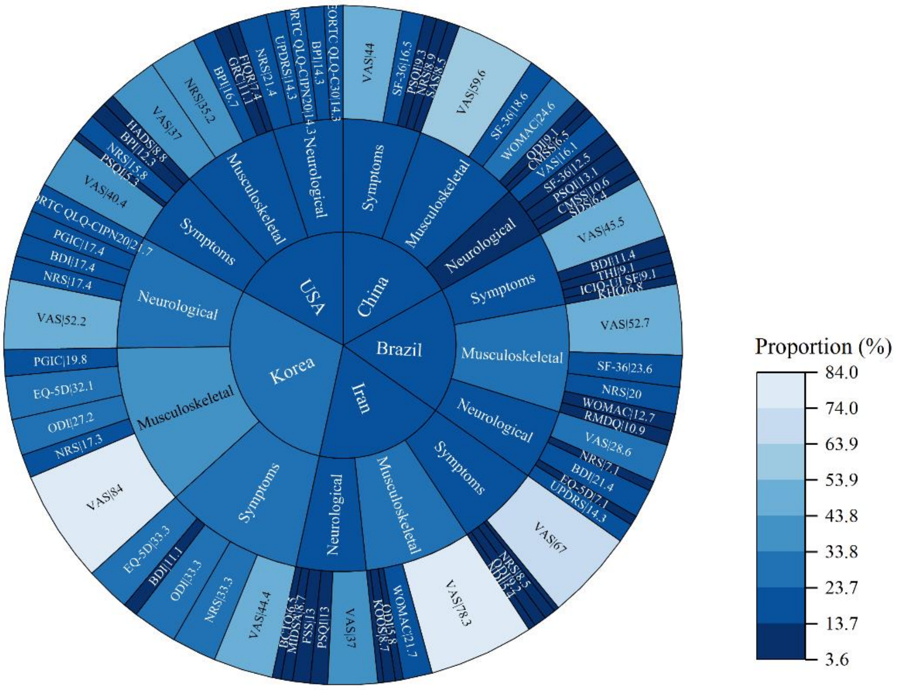

Abbreviations: VAS, Visual Analogue Scale; NRS, Numeric Rating Scale; BDI, Beck Depression Inventory; PSQI, Pittsburgh Sleep Quality Index; ODI, Oswestry Disability Index; SF-36, Short-Form 36-item Health Survey; EQ-5D,EuroQoL 5-Dimension 5-Level; BPI, Brief Pain Inventory; CMSS, Chinese Medicine Syndrome Score; WOMAC, The Western Ontario and McMaster Universities Osteoarthritis Index; EORTC QLQ-CIPN20, EORTC Quality of Life - Chemotherapy-Induced Peripheral Neuropathy; FSFI, Female Sexual Function Index; MPQ, McGill Pain Questionnaire; PGIC, ; SAS, Self-rating Anxiety Scale; SDS, Self-rating Depression Scale; STAI, State-Trait Anxiety Inventory; UPDRS, Unified Parkinson’s Disease Rating Scale; BCTQ, Boston Carpal Tunnel Questionnaire; DASH, Disabilities of Arm, Shoulder and Hand Questionnaire; DRSP, Daily Record of Severity of Problem; EHP-5, Endometriosis Health Profile-5; EORTC QLQ-C30, EORTC Quality of Life Questionnaire - Core Questionnaire; FIQR, Fibromyalgia Impact Questionnaire; FSS, Fatigue Severity Scale; GRC, Global Ratings of Change Scale; HADS, Hospital Anxiety and Depression Scale; ICIQ-UI SF, International Consultation on Incontinence Questionnaire - Urinary Incontinence Short Form; ISI, Insomnia Severity Index; KHQ, King's Health Questionnaire; KOOS, Knee injury and Osteoarthritis Outcome Score; MIDAS, Migraine Disability Assessment; NDI, Neck Disability Index; RMDQ, Roland-Morris Disability Questionnaire; TCMSS, TCM Symptom Score; THI, Tinnitus Handicap Inventory.
